# Using Distributed Data over HBase in Big Data Analytics Platform for Clinical Services

**DOI:** 10.1155/2017/6120820

**Published:** 2017-12-11

**Authors:** Dillon Chrimes, Hamid Zamani

**Affiliations:** ^1^Database Integration and Management, IMIT Quality Systems, Vancouver Island Health Authority, Vancouver, BC, Canada V8R 1J8; ^2^School of Health Information Science, Faculty of Human and Social Development, University of Victoria, Victoria, BC, Canada V8P 5C2

## Abstract

Big data analytics (BDA) is important to reduce healthcare costs. However, there are many challenges of data aggregation, maintenance, integration, translation, analysis, and security/privacy. The study objective to establish an interactive BDA platform with simulated patient data using open-source software technologies was achieved by construction of a platform framework with Hadoop Distributed File System (HDFS) using HBase (key-value NoSQL database). Distributed data structures were generated from benchmarked hospital-specific metadata of nine billion patient records. At optimized iteration, HDFS ingestion of HFiles to HBase store files revealed sustained availability over hundreds of iterations; however, to complete MapReduce to HBase required a week (for 10 TB) and a month for three billion (30 TB) indexed patient records, respectively. Found inconsistencies of MapReduce limited the capacity to generate and replicate data efficiently. Apache Spark and Drill showed high performance with high usability for technical support but poor usability for clinical services. Hospital system based on patient-centric data was challenging in using HBase, whereby not all data profiles were fully integrated with the complex patient-to-hospital relationships. However, we recommend using HBase to achieve secured patient data while querying entire hospital volumes in a simplified clinical event model across clinical services.

## 1. Introduction 

Large datasets have been in existence, continuously, for hundreds of years, beginning in the Renaissance Era when researchers began to archive measurements, pictures, and documents to discover fundamental truths in nature [1–4]. The term “Big Data” was introduced in 2000 by Francis Diebold, an economist at the University of Pennsylvania, and became popular when IBM and Oracle adopted it in 2010 and thereafter in healthcare [5]. Gantz and Reinsel [6] predicted in their “The Digital Universe” study that the digital data created and consumed per year will reach 40,000 Exabyte by 2020, from which a third will be processed using Big Data technologies. Big Data has been characterized in several ways: as NoSQL key-indexed [7, 8], unstructured [9] computer interpretations, text, information-based [10], and so on. With this in mind, Big Data Analytics (BDA) in healthcare requires a more comprehensive approach than traditional data mining; it calls for a unified methodology to validate new technologies that can accommodate the velocity, veracity, and volume capacities needed to facilitate the discovery of information across all healthcare data types of healthcare domains [11].

There are many recent studies of BDAs in healthcare defined according to many technologies used, like Hadoop/MapReduce [12, 13]. BDA itself is the process used to extract knowledge from sets of Big Data [14]. The life sciences and biomedical informatics have been among the fields most active in conducting BDA research [15]. Kayyali et al. [16] estimated that the application of BDA to the US healthcare system could save more than $300 billion annually. Clinical operations and research and development are the two largest areas for potential savings: $165 billion and $108 billion, respectively [17].

Research has focused mainly on the size and complexity of healthcare-related datasets, which includes personal medical records, radiology images, clinical trial data submissions, population data, and human genomic sequences (Table 1). Information-intensive technologies, such as 3D imaging, genomic sequencing, and biometric sensor readings, are helping fuel the exponential growth of healthcare databases [12, 18]. Furthermore, the use of Big Data in healthcare presents several challenges. The first challenge is to select appropriate statistical and computational method(s). The second is to extract meaningful information for meaningful use. The third is to find ways of facilitating information access and sharing. A fourth challenge is data reuse, insofar as “massive amounts of data are commonly collected without an immediate business case, but simply because it is affordable” [19]. Finally, another challenge is false knowledge discovery: “exploratory results emerging from Big Data are no less likely to be false” [5] than reporting from known datasets. In cancer registries, for example, biomedical data are now being generated at a speed much faster than researchers can keep up with using traditional methods [20].

Certain improvements in clinical care can be achieved only through the analysis of vast quantities of historical data, such as length-of-stay (LOS); choice of elective surgery; benefit or lack of benefit from surgery; frequencies of various complications of surgery; frequencies of other medical complications; degree of patient risk for sepsis, MRSA,* C. difficile*, or other hospital-acquired illness; disease progression; causal factors of disease progression; and frequencies of comorbid conditions. In a study by Twist et al. [21], the BDA-based genome-sequencing platform Constellation was successfully deployed at the Children's Mercy Hospital in Kansas City (Missouri, US) to match patients' clinical data to their genome sequences, thereby facilitating treatment [22]. In emergency cases, this technology allowed the differential diagnosis of a genetic disease in neonates to be made within 50 hours of birth. Improvement of the platform using Hadoop reduced the time required for sequencing and analysis of genomes from 50 to 26 hours [23]. Therefore, a real-time diagnosis via BDA platform in healthcare to analyze hospital and patient data was successfully implemented. Yet, Chute [24] points out that health informatics are biased towards the classification of data as a form of analytics, largely, in the case in Canada, because the data standards of the DAD are primarily set by CIHI for clinical reporting. Proprietary hospital systems also have certain data standards that are partly determined by the physical movement of patients through the hospital rather than just diagnoses and intervention recordings.

Healthcare and hospital systems need BDA platforms to manage and derive value. The conceptual framework for a BDA project in healthcare, in essence of its functionality, is not totally different from that of conventional systems. Healthcare analytics is defined as a set of computer-based methods, processes, and workflows for transforming raw health data into meaningful insights, new discoveries, and knowledge that can inform more effective decision-making [25]. Data mining in healthcare has traditionally been linked to knowledge management, reflecting a managerial approach to the discovery, collection, analysis, sharing, and use of knowledge [26, 27]. Thus, the Discharge Abstract Database (DAD) and Admission Discharge Transfer (ADT) datasets are designed to enable hospitals and health authorities to apply knowledge from ad hoc data recording patient numbers, health outcomes, length-of-stay (LOS), and so forth [28]. And such a combination of ADT and DAD in real time could better link the movement and medical services of inpatients with treatments and diagnoses.

### 1.1. Study Objective

The objective was to establish an interactive and dynamic framework with front-end and interfaced applications (i.e., Apache Phoenix, Apache Spark, and Apache Drill) linked to the Hadoop Distributed File System (HDFS) and backend NoSQL database of HBase to form a platform with Big Data technologies to analyze very large data volumes. By establishing a platform, challenges of implementing and applying it to healthcare scenarios for clinical services could be validated by users to visualize, query, and interpret the data. The overall purpose was a proof of concept of Big Data capabilities to stakeholders, including physicians, VIHA administrators, and other healthcare practitioners. One working hypothesis was that NoSQL database created using hospital and patient data in differentiated fields would accurately simulate the patient data. Another hypothesis was that high performance could be achieved by using a few nodes optimized at the core CPU capacity and, therefore, used for clinical services. Lastly, patient data could be secured from configurations and deployment of HBase/Hadoop architecture and heavily relied on WestGrid's High Performance Computing (HPC). These hypotheses are related to five specific challenges: data aggregation, maintenance, integration, analysis, and pattern interpretation of value application for healthcare [28, 29].

Legality and ethics are a major contender to deal with within the realm of utilization of large datasets of patient data in healthcare [30]. Legislation mandates security, confidentiality, and privacy of patient data. The Health Insurance Portability and Accountability Act (HIPAA), as well as Freedom of Information and Protection of Privacy Act (FIPPA), requires the removal of several types of identifiers, including any residual information of patients [31]. These privacy legislations are a major barrier; however, privacy concerns can be overcome by using newer technologies, such as key-value (KV) storage services with somewhat advanced configurations and technical knowledge for ongoing operational access and maintenance. For example, Pattuk et al. [32] proposed a framework for securing Big Data management involving HBase, called Big Secret, that securely processes encrypted data over public KV stores. Hence, one method of ensuring patient data privacy/security is to use indexes generated from HBase, which can securely encrypt KV stores [8, 33, 34] with HBase further encryption with integration with Hive [35].

## 2. Methods 

In a hospital system, such as for the Vancouver Island Health Authority (VIHA), the capacity to record patient data efficiently during the processes of ADT is crucial for timely patient care and the enhanced patient-care deliverables. The ADT system is referred to as the source of truth for reporting of hospital operations from inpatient to outpatient and discharged patients. Among these deliverables are reports of clinical events, diagnoses, and patient encounters linked to diagnoses and treatments. Additionally, in Canadian hospitals, discharge records are subject to data standards set by Canadian Institute of Health Information (CIHI) and administered into Canada's national DAD repository. Moreover, ADT reporting is generally conducted through manual data entry to a patient's chart and then it is combined with Electronic Health Record (EHR) (adding to further complications of possibly compromising autopopulated data) that might consist of other hospital data in reports to provincial and federal health departments [36]. A suitable BDA platform for a hospital should allow integration of ADT and DAD records and to query that combination to find trends at its extreme volumes.

### 2.1. Big Data Technologies and Platform Services

Big Data technologies fall into four main categories: high performance computing, data processing, storage, and resource/workflow allocator, like Hadoop/MapReduce [37–41] (Table 2). A high performance computing (HPC) system is usually the backbone framework of a BDA platform, for example, IBM's Watson and Microsoft Big Data solutions [42]. An HPC system consists of a distributed system, grid computing, and a graphical processing unit (GPU).

A distributed computing system can manage hundreds of thousands of computers or systems, each of which is limited in its processing resources (e.g., memory, CPU, and storage). By contrast, a grid computing system makes efficient use of heterogeneous systems with optimal workload management servers, networks, storage, and so forth. Therefore, a grid computing system supports computation across a variety of administrative domains, unlike a traditional distributed computing system. Furthermore, a distributed Hadoop cluster, with its distributed computing nodes and connecting Ethernets, runs jobs controlled by a master. “Hadoop was first developed to fix a scalability issue affecting* Nutch*, an open-source crawler and search engine that uses the MapReduce and* BigTable* methods developed by Google” [19]. Distributed computing using MapReduce and Hadoop represents a significant advance in the processing and utilization of Big Data in healthcare [25, 40].

Considering the design and implementation of BDA systems for clinical use, the basic premise is to construct a platform capable of compiling diverse clinical data. However, the process of Ethics and Research Capacity at VIHA for approval for the entire patient data of the hospital system was not possible. Secondly, it was not possible to piece together summarized data specific to health outcomes because this data has already been summarized. Thirdly, real data in the data warehouse at VIHA will require several months to review and develop the solution to use Big Data technologies. Lastly, performance benchmarking of the platform needs to be determined with the current data query tools and workflow at VIHA, which means that simulation at extremely large volume can prove to be of high performance and usability. Therefore, the study focused on simulation conducted with VIHA's real metadata and exchanged knowledge on how the ADT and DAD could be used in production.

### 2.2. Healthcare Big Data Analytics Framework

Hadoop/MapReduce framework was proposed to implement HBDA and analyze emulated patient data over a distributed computing system that is not currently used in acute patient-care settings at VIHA and other health authorities in British Columbia, Canada. The teamed collaboration between UVic, Compute Canada/WestGrid, and VIHA established the framework of the HBDA platform. It comprised innovative technologies like the Hadoop HDFS with MapReduce programming and a NoSQL database. The HBase database construct was complex and had many iterations of development over the past three to four years. HBase is an open-source, distributed key-value (KV) store based on Google's* BigTable* [43]—persistent and strictly consistent NoSQL system using HDFS for data storage. Furthermore, with all these technical components to construct the platform, the build also considered the workflow at VIHA with their respective clinical reporting workgroups with the same metadata from real hospital datasets.

The functional platform was tested for performance of data migrations or ingestions of HFiles via Hadoop (HDFS), bulkloads of HBase, and ingestions of HFiles to Apache Spark and Apache Drill. In this study performances were proof-of-concept testing using simulated data with the same replicated metadata and very large volume. Furthermore, this study involved six Hermes cores (each core has 12 Computer Processing Units (CPU) cores). These CPUs accounted for only a total of 72 cores out of the overall maximum of 4416 cores available at WestGrid-UVic. There were many configurations and package components to include in the build, such as Apache Phoenix, Apache Spark, and Apache Drill, as well as Zeppelin and Jupyter Notebook interfaces.

### 2.3. Replication, Generation, and Analytics Process

Metadata is information about the data that is established in a system as a structured standard to record and retrieve information accurately. It is the structure of metadata that allows for data profiles (i.e., characteristics, sources, and character lengths) to be established in a database. And in healthcare this means data is standardized effectively for patient records to be accurate when retrieved or viewed in an EHR. In the case of VIHA, the metadata of the ADT system allows for patient data to be recorded when a patient is admitted to the hospital, assigned to a bed, and provided other medical services. The structure itself is not proprietary to the system and does not contain any real patient data. In the meetings, with VIHA personnel, the core metadata of ADT/DAD were verified with questions scripted for the three main groups (Box 1). With the help from health professionals and providers, their current fast and reliable queries were revealed, and unknown and desired health trends, patterns, and associations of medical services with health outcomes were unveiled. The records comprise patient demographics, emergency care, ADT, clinical activities, diagnoses, and outcomes information.

To accomplish these objectives, Island Health's partial core metadata from ADT/DAD systems was obtained via knowledge transfer in interviews with specific teams working at Royal Jubilee Hospital (RJH) and Nanaimo Regional General Hospital (NRGH). Knowledge transfer with VIHA personnel and current reporting limitations were documented, recorded, and verified in summary after several meeting iterations.

Information from the informatics architecture team was composed of DAD dictionary and the selected data elements. Information on metadata and the frequencies of three core data elements (i.e.,* Admin Source, Admin Type, *and* Encounter Type*) from the BI Data Warehouse team will be the ADT system database and the core data elements it comprises. Information from Information Specialist and Clinical Information Support will be the metadata relationship between ADT and DAD at VIHA. Clinical reporting works with Cerner Person Management tools and Med2020 WinRec Abstracting on organizing of the metadata before it is stored in a data warehouse. VIHA's privacy/security team was also interviewed on data ownership and necessary steps to get approval when using real data that might require public disclosure.

Metadata was set at over 90 columns and randomized based on data dictionary examples and from VIHA interviews. For example, metadata for the diagnostic column was set with standardized metadata of International Classification of Disease version 10 Canadian or ICD-10-CA codes, and personal health number (PHN) has ten numerical digits while the patient's medical record number (MRN) for that encounter has nine numerical digits. All data elements and their required fields, as well as primary and dependent keys, were recorded for completed trials of the necessary columns to generate the emulation of aggregated hospital data. The generator included all important data profiles and dependencies were established through primary keys over selected columns (Table 3).

At VIHA, health informatics architecture has direct relation to the DAD abstracting, as it is a manual process and dependent on* Admit Type* and* Admit Source* obtained from Cerner's hospital system. The emergency system is separate from the ADT, and there are also planned procedures in the triage that are not part of the ADT system. Doctors and Nurses refer to the patient encounter as the source of “truth” of patient encounters in the ADT system. Each patient can have multiple encounter numbers with overall one million encounters annually registered at VIHA. In contrast, DAD is an attribute of the encounter, mostly diagnosis and discharge, while ADT represents a person's relationship to the hospital system with medical services and patient location(s). However, this study did include patient movement in hospital (which is currently not queried at large levels) and patient transfers. A transfer is a change in the encounter that is not always represented by digital documentation; for example, a patient may be transferred to NRGH in Nanaimo and then receive a new encounter after being discharged from RJH, and vice versa.

The data warehouse team working with health professionals for clinical reporting can rely on comma-separated value (.*csv*) formats when importing and exporting data. Therefore, this study opted to use the ingested .*csv* files directly for analytics instead of HBase, which had previously been used on this platform along with Apache Phoenix and its SQL-like code [44]. Three data sizes (50 million and one and three billion records) were used as benchmark checks of how different packages (Apache Spark and Drill) scaled with data size for clinical use.

It is important to note that this study is about performance testing of ADT/DAD queries of a distributed filing system (Apache-Hadoop) with a processing (MapReduce) configuration on an emulated NoSQL database (HBase) of patient data. The platforms tested the totally randomized generated data with replicated duplicates for every 50 million patients' encounters with that of replicated groupings, frequencies, dependencies, and so on in the queries. The pipelined process included five stages or phases that coincided with the challenges outlined in Section 1 and the overall study's objectives.

#### 2.3.1. Data Acquisition

*(a) Data Emulation Using HBase. *In the emulated dataset, each row represented encounter-based patient data, with diagnoses, interventions, and procedures specific to that patient, that the current ADT system has in its database schema linked to a bigger data warehouse (refer to Table 3 for clinical cases). This patient-specific structure in the database allowed for active updates for accurate patient querying over the platform, simulated throughout the lifetime of that person. Chawla and Davis [33] showed that utilization of ICD diagnosis codes over a patient-centered framework allowed for a seamless integration with a variety of data from electronic healthcare systems with patient-centric ADT; this method could accurately query readmission rates and quality of care ratings and demonstrate meaningful use and any impact on personal and population health. Therefore, the platform used a similar methodology to establish the structure of the data model of combining encounter-based ADT with standardized diagnosis; every encounter has a separate diagnosis, procedures, and most responsible provider.

All necessary data fields were populated for one million records before replication to form one and three billion records. The recorded workflow provided a guideline to form the NoSQL database, as a large distributed flat file. The patient-specific rows across the columns according to the existing abstraction were further emulated; HBase established a wide range of indexes for each unique row, and each row contained a key value that was linked to the family of qualifiers and primary keys (columns). The HBase operations were specific to family qualifiers at each iteration; therefore, the data was patient-centric combined with certain DAD data (from different sources of metadata) in the rows and columns, such that summary of diagnosis or medical services could be queried.

*(b) Data Translation. * Since the performance tests of queries on the platform relied on data emulation, as a proof of concept, the usual high-speed file transfer technologies (such as SCP and GridFTP) were used to transfer data to the HPC parallel file system (GPFS). When the pharmaceutical data was ingested on the Hadoop/MapReduce framework, it showed the same results as benchmarked data. The Hadoop and HBase were then used as NoSQL database bulkload utilities to ingest the data. To establish data structure, the* EncounterID* was set as a Big Data integer (so that it can reach billions of integers itemized sequentially without limitation) and indexed based on that integer via HBase for each unique row at every iteration that followed. This indexed-value column, unique for every row, causes MapReduce to sort the KV stores for every one of the iterations that can increase the integrity of the data and increase its secured access once distributed.

#### 2.3.2. Data Maintenance and Troubleshooting

The emulated data was stored and maintained in the HPC parallel file (~500 GB) and over the BDA platform under HDFS. The replication factor for HDFS was set to three for fault tolerance. The large volume of datasets was reutilized to test the performance of different use cases or queries conducted by the analytics platform. This required innovation, in an agile team setting, to develop stages in the methodology unique to BDA configurations related to healthcare databases.

#### 2.3.3. Data Integration (Clinical)

This step was very important because the SQL-like Phoenix queries had to produce the same results as the current production system at VIHA. All results were tested under a specific data size and comparable time for analysis, whether the query was simple or complex. The data results also had to show the exact same columns after the SQL-like queries over the constraint of the family qualifiers (as primary keys). Over a series of tests, certain columns were included or excluded as qualifiers in the SQL code for constraints. Once the results were accurate and were the same as those benchmarked, those qualifiers remained for each of the iterations run via Hadoop, to generate the one billion totals.

#### 2.3.4. Data Analysis

In this step, the study conducted a proof-of-concept analysis of task-related use cases specific to clinical reporting. The queries were evaluated based on the performance and accuracy of the BDA framework over the one billion rows. For example, a task-based scenario for the analysis included the following.

*(a) Analysis Questions/Scenarios. *A typical analysis scenario was as follows: clinicians suspect that frequent movement of patients within the hospital can worsen outcomes. This is especially true in those who are prone to confusion due to changes in their environment (i.e., the elderly).

*(b) Analytic Algorithms and Tools. *To handle intensive computation, simplified algorithms were applied and distributed over database nodes. For instance, there was some default MapReduce-based a priori data-mining algorithm to find associated patterns in the dataset. The customized MapReduce templates were tailored to be used via Phoenix (later, in a separate part of this study, similar algorithms were also tested via Apache Spark and Apache Drill) on the HBase database nodes. For developing some of the software pipeline, the plan was to establish and engineer alternative products with Spark such as Jupyter and Zeppelin to work over Hadoop and establish a query GUI interface to interactively run all test queries simultaneously and display all durations to generate results. Apache Drill was also selected because the same queries tested in Phoenix and Spark can be used plus its interface can be integrated over Hadoop.

*(c) Pattern Validation and Presentation. *The study undertook more than five phases of the configuration process (over several months and years) to query the data distributed. The initial aim of assessing how well the models will perform against a large dataset was first carried out with publicly available annual (2005-2006) inventory of pharmaceuticals (~5 MB). Once the pharmaceutical data ingested on the Hadoop/MapReduce framework showed the same results benchmarked. Simulated queried results from the platform were to follow the DAD reporting for health outcomes at the hospital level and each row was deemed to represent one patient encounter. For this to succeed, domain experts and physicians were involved in the validation process and interpretation of the results and end users' usability of the query tools. Since the data was randomized at one million records and replicated iteratively at 50 million to one billion and then to three billion, the data results were already known beforehand; therefore, the trends detected will be randomized data clusters only.

#### 2.3.5. Data Privacy Protection

The established framework of the platform used WestGrid's existing security and the privacy of its supercomputing platform while reviewing and identifying regulations for eventually using real patient data over the platform (external to the hospital's data warehouse). The following method was applied, which included four steps.

**Step 1 .**

HBase creates indexes for each row of data that cannot be queried with direct access, and queries can only be generated when accessing the deployment manager (DM) on the platform. That is, the data cannot be viewed at all by anyone at any time or for any duration; only queries can show the data that is HBase-specific and nonrecognizable without Hadoop and HBase running, as well as the correct scripts to view it.

**Step 2 .**

Executing data replication, as a generator over the platform, worked in conjunction with business/security analysts to identify the masking or encryption-required algorithms that represented optimal techniques to replace the original sensitive data.

**Step 3 .**

Review was carried out with the related regulations regarding privacy protection regulations and principles, such as the HIPPAA, Freedom of Information and Protection of Privacy Act (FIPPA), Personal Information Protection Act (PIPA), and the use of the public KV stores established in semipermanent databases of HBase distributed by Hadoop.

**Step 4 .**

Test of the replicated dataset was executed by an application process to test whether the resulting masked data could be modified to view. A real dataset (large annual inventory of pharmaceuticals) was tested and verified firstly, since studies have shown that the distribution of data using Hadoop has many inherent processes that restrict access to running ingestions [43, 44].

### 2.4. Implementing Framework for Clinical Use

In this section, the steps and experiences implementing the technical framework and application of a BDA platform are described. The established BDA platform will be used to benchmark the performance of end users' querying of current and future reporting of VIHA's clinical data warehouse (i.e., in production, spanning more than 50 years of circa 14 TB). To accomplish this, Hadoop environment (including the Hadoop HDFS) from a source was installed and configured on the WestGrid cluster, and a dynamic Hadoop job was launched.

The construction and build of the framework with HBase (NoSQL) and Hadoop (HDFS) established the BDA platform. This construct coincided with and is enforced by the existing architecture of the WestGrid clusters at UVic (secure login via LDAP directory service accounts to deployment database nodes and restricted accounts to dedicated nodes). It was initially running the architecture of the platform with five worker nodes and one master node (each with twelve cores) and planned to increase the (dedicated) nodes to eleven and possibly to 101, as well as incorporating a nondedicated set of virtual machines on WestGrid's OpenStack cloud.

The queries via Apache Phoenix (version 4.3.0) resided as a thin SQL-like layer on HBase. The pathway to running ingestions and queries from the build of the BDA platform on the existing HPC was as follows:

  .*csv* flat files generated → HDFS ingestion(s) → Phoenix bulkloads into HBase → Apache Phoenix queries.

This pathway was tested in iteration up to three billion records (once generated) for comparison of the combination of HBase-Phoenix versus Phoenix-Spark or an Apache Spark Plugin (Apache Phoenix, 2016), under this sequence and after loading the necessary module environments for Hadoop, HBase, and Phoenix and testing initial results linked to the family qualifiers and HBase key-value entries [28, 29].

Performance was measured with three main processes: HDFS ingestion(s), bulkloads to HBase, and query times via Phoenix. One measurement of ingestion time in total for iterations and overall was established to achieve the total desired number of records, that is, one billion and three billion from 50 million replicated [29]. We also computed the ingestion efficiency (IE) and query efficiency (QE) of one billion compared to 50 million records using the following formula:

(1) $$\begin{array}{c} IE,QE=\frac{1\left(3\right)B\times T_{i}\left(50M\right)}{50M\times T_{i}\left(1\left(3\right)B\right)}, \end{array}$$

where *T*_*i*_(*N*) is the time it takes to ingest *N* records to either HDFS or HBase.

**Figure 1:**
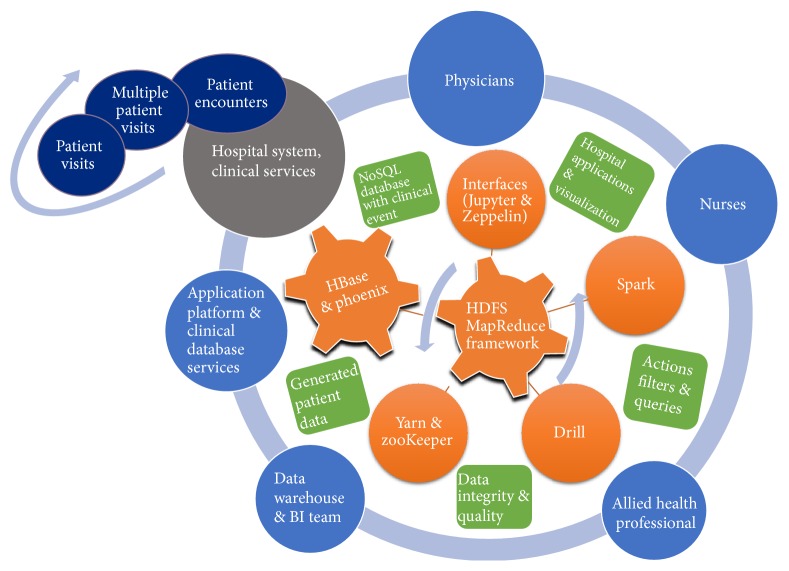
Big Data Analytics (BDA) platform designed and constructed as patient encounter database of hospital system.

Apache Spark (version 1.3.0) was also built from source and installed to use on HBase and the Hadoop cluster. The intent was to compare different query tools like Apache Spark and Drill, implemented over the BDA platform, against Apache Phoenix using similar SQL-like queries. The entire software stack used in the platform has at its center HDFS (Figure 1).

## 3. Results 

Data profiles, dependencies, and the importance of the metadata for reporting performance were also emulated and verified. Current reporting limitations were recorded if the combinations of the DAD and ADT were done in one distributed platform running parallel queries. A total of 90 columns were confirmed as important to construct necessary queries and to combine ADT data with DAD data in the Big Data platform. Additionally, the queries derived were compared with clinical cases and how that interacted with the performance of the platform was representative of the clinical reporting at VIHA.

### 3.1. Technical Implementation

HBase (NoSQL version 0.98.11) was composed of the main deployment master (DM) and failover master, the* RegionServers* holding HBase data, and a ZooKeeper of five nodes to orchestrate the ensemble, called* RegionServers. *HBase consists of unique rows and each row contains a key value. A key-value entry has five parts: row-key* (row)*, family* (fam)*, qualifier* (qua)*, timestamp* (ts)*, and value* (val)* denoted as KEY≔*row*||*fam*||*qua*||*ts* [28]. Additionally, to establish the HBase key-value entries, there are four operations:

1. *put*, which inserts data
2. *get*, which retrieves data of a specific row
3. *delete*, which removes a data row
4. *scan*, which retrieves a range of indexed rows.

The steps carried out to run Hadoop modules are shown in Box 2.

The platform worked as expected after modified configurations of Hadoop's* hdfs-site.xml.* Additionally, the number of replicas was set to three in the xml with connection to InfiniBand or* ib0*. To interact with HDFS, command scripts were run to automate the ingestion step (generating data replication in the exact format specified by SQL script to the nodes).

**Figure 2:**
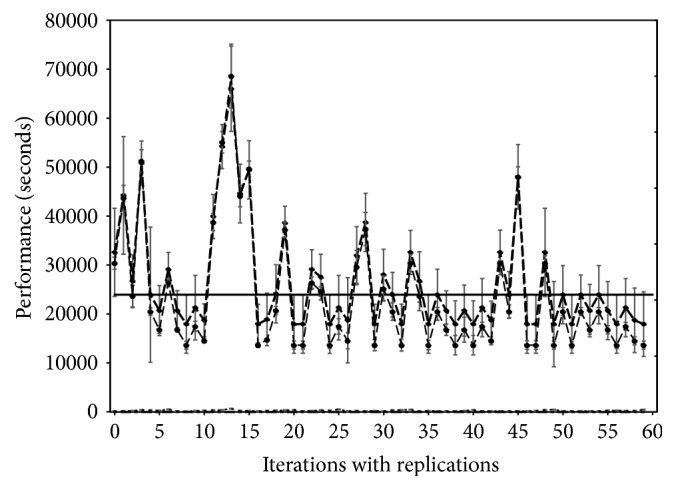
Performance (seconds) of 60 ingestions (i.e., 20 replicated 3 times) from Hadoop HDFS to HBase files, MapReduce indexing, and query results. Dashed line is total ingestion time and the dotted line is time to complete the Reducer of MapReduce. The bottom dashed-dot lines are the times to complete Map of MapReduce and the duration (seconds) to run the queries.

The Map part of MapReduce on the platform showed high performance at 3–10 minutes, but the Reduce took 3–12 hours (Figure 2). Apache Phoenix (version 4.3.0), a thin layer on top of HBase, was used as ingestion structured file and schema-based data into the NoSQL database.

To improve the ingestion of the one billion rows and 90 columns to attempt to generate 1–10 billion rows, local hard disks of 40 TB in total were physically installed on the worker nodes. After local disks were installed on five (worker) nodes, a set of shell scripts was used to automate the generation and ingestion of 50 million records at each of the iterations via MapReduce. The maximum achieved was 3 billion due to operational barriers, workflow limitations, and table space because key stores almost tripled the amount of space used for each of the ingestions (Table 4). In total, including all the testing, about 6–9 billion rows were ingested to the local disks in iteration of which three billion were correctly indexed and could be accurately consistently queried.

**Figure 3:**
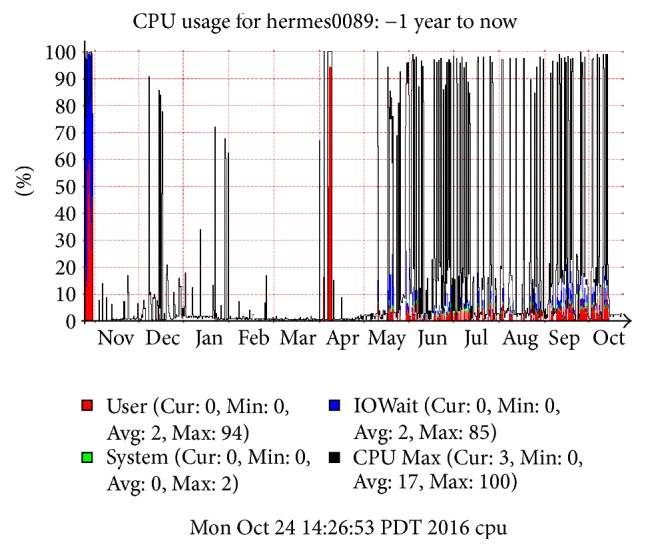
A year of varied iteration and CPU usage (at 100%) on Hemes89 node reported from WestGrid showing variation in the duration of the ingestion of 50 million records over each of the iterations. The graph shows the following: user (in red), system (in green), IOWait time (in blue), and CPU Max (black line).

Other findings of Big Data technology limitations installed on WestGrid's architecture were ongoing manual intervention (over three-five months) which was required to constantly fine-tune the performance of bulkloads from MapReduce to HBase. Hadoop had ingestions exhibiting high performance, for circa three minutes to complete task for 258 MB or each 50 million rows. Sometimes HDFS was unbalanced and had to be rerun to rebalance the data to the nodes or when the local disk at 500 GB did not failover to 2 TB disks installed, the entire ingestions had to start all over again because HBase could not reindex them, and, therefore, its queries were invalid with no indexes, which drastically slowed performance when not operational. There were some findings on optimized performance of the platform. CPU usage needs to be maxed, which is during mid-May to October 2016; it pinged at 100% but did not stay due to running compaction after each of the ingestions took over 4 hours (Figure 3). And, the IO disk usage needs to reach the best possible throughput provided or closest to 100% CPU, which showed 160 MB/s was achieved and pinged at approximately the same time at the peak performance of the corresponding ingestions.

### 3.2. Clinical Analytics and Visualizations

The deployment of the Hadoop environment on the nodes was carried out behind the backend database scenes via a sequence of setup shell scripts that the user can then adjust configurations to match the needs of the job and its performance. There were 22 SQL-like queries tests for querying reports, instances, and frequencies in the ADT/DAD data over the 50 million to 1–3 billion rows. Ten queries were categorized as simple while others were complex; these included more than three columns and three primary keys across the 90 possible columns. All queries, simple (linear) and complicated (exponential and recursive), were less than two seconds for one billion and almost the same for three billion when the nodes were, eventually, balanced by Hadoop; however, some queries were more than three seconds and less than 4 seconds for three billion with unbalanced nodes. There were no significant differences between simple and complex query types and possible two-second increase when nodes were unbalanced. Caching did not influence the query times. There was no significant difference in the performance of simple versus complex queries. The performance speed, even at one to three billion rows for complex queries, was extremely fast compared to the 50 million rows queried. It did require months of preparation to get to the task of testing the platform with the queries. Health data that was involved with hospital outcomes and clinical reporting was combined to form a database and distributed over nodes as one large file, up to 30 TB for HBase. All the pertinent data fields and much more were used.

The results showed that the ingestion time of one billion records took circa two hours via Apache Spark. Apache Drill outperformed Spark/Zeppelin and Spark/Jupyter [29]. However, Drill was restricted to running more simplified queries and was very limited in its visualizations that exhibited poor usability for healthcare. Zeppelin, running on Spark, showed ease-of-use interactions for health applications, but it lacked the flexibility of its interface tools and required extra setup time and 30-minute delay before running queries. Jupyter on Spark offered high performance stacks not only over the BDA platform but also in unison, running all queries simultaneously with high usability for a variety of reporting requirements by providers and health professionals.

**Figure 4:**
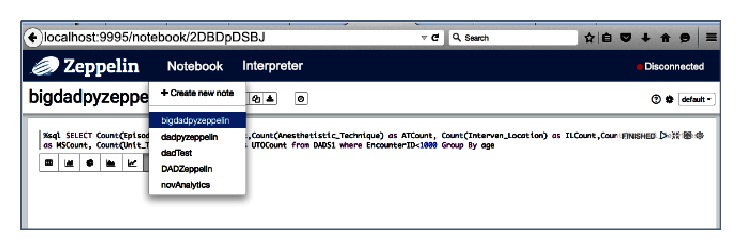
Zeppelin interface with Apache Spark with multiple notebooks that can be selected by clinical users.

Drill did perform well compared to Spark, but it did not offer any tools or libraries for taking the query results further. That is, Drill proved to have higher performance than Spark but its interface had fewer functionalities. Moreover, algorithms (as simple as correlations between different columns) were time-demanding if not impossible to express as SQL statements. Zeppelin, on the other hand, offered the ability to develop the code, generate the mark-down text, and produce excellent canned graphs to plot the patient data (Figure 4). Combined with the richness of Spark and* Pyspark*, Zeppelin provided a canned visualization platform with graphing icons. The plots under Zeppelin, however, are restricted to the results/tables obtained from the SQL statements. Furthermore, the results that were produced directly from the Spark SQL context did not have any visualization options in Zeppelin. Generating results from queries via Zeppelin took much longer (over 30 minutes). Establishing the platform to run queries on the interface and generate results via Zeppelin took longer than Jupyter [29].

**Figure 5:**
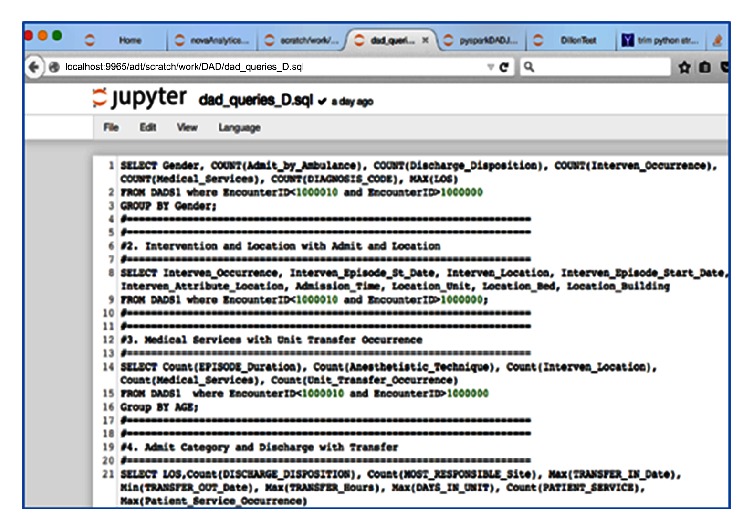
Spark with Jupyter and SQL-like script to run all queries in sequence and simultaneously.

**Figure 6:**
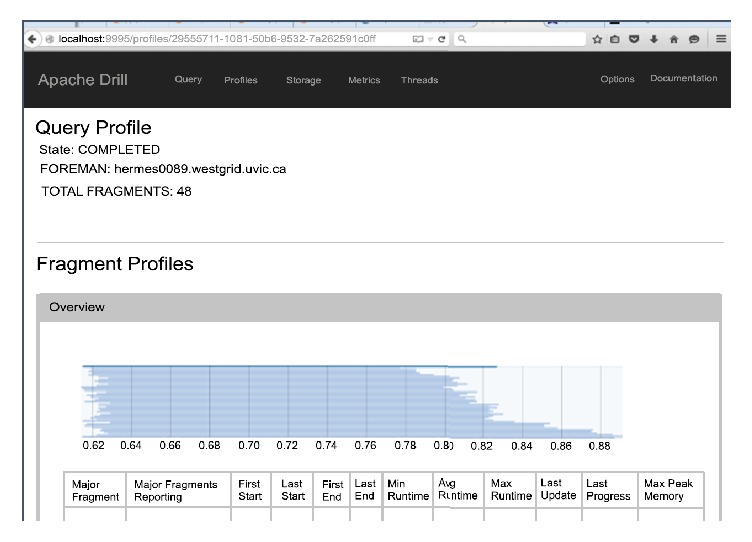
Drill interface customized using the distributed mode of Drill with local host and running queries over WestGrid and Hadoop.

With Jupyter, more configurations with the data queries were tested. It exhibited similar code to ingest the file (Figure 5), the same Spark* databricks* initialized in the interface, and its SQL to query as Zeppelin, but at the expense of writing the visualization code, using the* matlplotlib* Python package in addition to other powerful tools, such as Pandas, that is, a powerful Python data analysis toolkit. The local host was added to Hermes node to access Jupyter via the BDA platform to compensate for the lack of visualization options via the Zeppelin interface. Jupyter supplied more visualization defaults and customization than Drill for its distributed mode and its interface to run the query (Figure 6) was severely lacking usability of any visualization tools.

## 4. Discussion

The ultimate goal of the study was to test the performance of the Big Data computing framework and its technical specifications cross platform against all challenges specific to its application in healthcare. This goal was accomplished by combining ADT and DAD data through ingestions over the Hadoop HDFS and the MapReduce programming framework. High performance over the BDA platform was verified with query times of less than four seconds for 3 billion patient records (regardless of complexity), showing that challenges of aggregation, maintenance, integration, data analysis, and interpretative value can be overcome by BDA platforms.

### 4.1. Modeling Patient Data of Hospital System

There are analytical challenges in many Canadian healthcare systems because of separated silos of aggregations. There are complex and unique variables that include “(1) information used; (2) preference of data entry; (3) services on different objects; (4) change of health regulations; (5) different supporting plans or sources; and (6) varying definition of database field names in different database systems” [45]. Big Data in healthcare can cover tens of millions or billions of patients and present unprecedented opportunities. Although data from such sources as hospital EHR systems are generally of much lower quality than data carefully collected by researchers investigating specific questions, the sheer volume of data may compensate for its qualitative deficiencies, provided that a significant pattern can be found amid the noise [14, 46]. Ultimately, it was designed not only to replicate data but to simulate the entire volume of production and archived data at VIHA, and possibly the Province of British Columbia, such that real patient data from hospitals will be approved to utilize the platform. Therefore, the messiness of the data and its influence on the simulation were not tested, although this could potentially affect accuracy and performance when querying real data.

The ADT data are very difficult to emulate because they are from Cerner System, which uses a kernel to create alias pools for ~1000 different tables in the database. Simply creating one flat file cannot emulate the complex metadata relationships and does not guarantee that the data are treated uniquely for each encounter row when the encounters can change over time or several are linked to the same patient. However, if the data is extracted from the automated hospital system and it is confirmed that the columns are correct with unique rows, it should be possible to combine it with DAD data with similar unique keys and qualifiers. The complex nature of HBase means that it is difficult to test the robustness of the data in emulations based on real data. Several steps were required to prepare the DAD database alone for statistical rendering before it was sent to CIHI. The actual columns used in this study are the ones used by VIHA to derive the information accurately in a relational database, which ensures the data is in alias pools and not duplicated for any of the encounters. Other research reviews (e.g., [5, 30, 47, 48]) have stressed the importance of patient data modeling with Big Data platforms in healthcare, indicating that a lack of BDA ecosystems is one of the reasons why healthcare is behind other sectors in utilizing current technologies to harness Big Data. Nelson and Staggers [5] noted that nursing informatics and data from nurse progress notes are underutilized in hospital systems. Wang et al. [47] also compare bioinformatics with healthcare and Big Data applications. Bioinformatics can match extremely large libraries of genetic data to libraries of medications or treatments; however, such matching cannot be performed at the scale of large hospital systems, and patient-centric frameworks and current traditional practices of storing relational data make it difficult to replicate for other database types, especially Big Data. Chawla and Davis [33] and Kuo et al. [48] argue that even structured data lack interoperability among hospital systems, so that no solutions could possibly link all data. At VIHA, for example, it is difficult to link the DAD and ADT data on encounters, because the DAD data on diagnosis and intervention are not stored together or integrated or have relational dependencies in an all-in-one data warehouse, while the ADT automatically links the data to encounters [5, 48]. Therefore, more validation is required to match corresponding medical services in ADT to patient diagnosis in that admission time and source.

It was more complicated to validate the simulated data in Spark and Drill with real data. Scott [49] indicated that the battlefield for the best Big Data software solutions is between Spark and Drill and that Drill can emulate complex data much more efficiently than Spark because Spark requires elaborate Java, Python, and Scala coding to do so. Nonetheless, both Spark and Drill were significantly faster than HBase in ingesting files directly into Hadoop via* Drillbits *(Drill) with ZooKeeper and MapReduce and RRD transformations with MapReduce (Spark). The tools used totally different processes across the nodes, and without indexing there is a lack of encrypted data (which patient data requires); those processes did, in the end, produce the same query, but that is because the platform was set to ingest the already-indexed files into Spark and Drill. Absence of indexing increases the risk of inaccuracies (even though the framework was more fault-tolerant when running Spark and Drill). Therefore, the Big Data tools and inherent technologies highly influence the clinical services of the data established and resulting data from queries.

Wang et al. [50] support this study's claim in their statement that nonrelational data models, such as the KV model, are implemented in NoSQL databases. Wang et al. [47] further stated that NoSQL provided high performance solutions for healthcare, being better suited for high-dimensional data storage and querying and optimized for database scalability and performance. A KV pair data model supports faster queries of large-scale microarray data and is implemented using HBase (an implementation of Google's BigTable storage system). The new KV data model implemented on HBase exhibited an average 5.24-fold increase in high-dimensional biological data query performance compared to the relational model implemented on MySQL Cluster and an average 6.47-fold increase on query performance on MongoDB [25]. The performance evaluation found that the new KV data model, in particular its implementation in HBase, outperforms the relational model currently implemented and, therefore, supports this study's NoSQL technology for large-scale data management over operational BDA platform of data from hospital systems.

### 4.2. HBase Database for Clinical Reporting

There are many alternative solutions for Big Data platforms; choice of the best solution depends on the nature of the data and its intended use (e.g., [51]). In practice, while many systems fall under the umbrella of NoSQL systems and are highly scalable (e.g., [51, 52]), these storage types are quite varied. However, each comes with its unique sets of features and value propositions [53]. For example, the key-value (KV) data stores represent the simplest model of NoSQL systems: they pair keys to values in a very similar fashion to how a map (or hash table) works in any standard programming language. Various open-source projects have been implemented to provide key-valued NoSQL database systems; such projects include Memcached, Voldemort, Redis, and Basho Riak [25]. Another category of NoSQL systems is document-oriented database stores. In these systems, a document is like a hash, with a unique ID field and values that may be any of a variety of types, including more hashes. Documents can contain nested structures, so they provide a high degree of flexibility, allowing for variable domains such as MongoDB and CouchDB [25]. These other categories could be used for hospital data; however, in this study HBase was chosen as the database type and technology because it simplified the emulation of the columns using the metadata in each column rather than the data types and the actual relationships among the data.

HBase also has a dynamic schema that can be uploaded via other Apache applications; therefore, the schema can be changed and tested on the fly. If HBase had not been used, more complex data models would have been needed to map over the Hadoop/MapReduce framework. Another benefit of using HBase is that further configurations can be accomplished for multirow transactions using a comma-separated value* (.csv)* flat file [51, 54]. Additionally, the longer these identifiers are, the bigger the KV of data storage in HBase will become; therefore, identifier length was standardized in this study as the minimum required depicting the data profile. Problems appeared while creating corresponding row keys in HBase. The ingestions were not evenly distributed, and the increasing keys in a single region may have contributed to the Reduce being slow [25].

Our study showed that compaction on HBase improved the number of successful runs of ingestion; however, it did not prevent failure of the nodes, a finding that is supported by other studies, (e.g., [39, 55–58]). However, the platform used in our study had ran into the problem of HBase's* RegionServer* hitting the InfiniBand correctly and fully, and the settings to run compaction after each ingestion did not always compact the files correctly, which caused the entire operational iteration of ingestion to fail.

### 4.3. HBase with Security/Privacy

In Canada, population health data policy relies on legislative acts for public disclosure of the data accessed externally outside health authority's boundaries [59]. Our BDA platform utilized existing architecture at WestGrid at UVic external to VIHA. WestGrid does maintain a secure environment for restricted access to accounts, and our Hadoop/HBase ingestion processes cannot be accessed by anyone other than the current authorized user. Thus, the BDA platform is highly secure. However, we showed that to replicate from source to HBase to form at least one billion required one week timeframe. Therefore, the data needs to be stored before running the queries, as Moselle [60] stated that if the data is stored with some permanency even over a few hours, public disclosure is required.

## 5. Limitations and Future Work

Advantage of using Apache Spark or Drill over Phoenix is less reliance on MapReduce, which speeds up performance; however, then there is major limitation of data not accurately representative of clinical events, and data is less encrypted. Therefore, there is a performance trade-off. A further limitation of this study was on linkage between the technologies and representations of the patient data for clinical use; HBase at large volumes did not achieve fully integrated complex hospital relationships. Without complete validation, the technologies cannot be certified by the health authority. More work on using key-value storage for BDA should be considered in simplified clinical event models across many clinical services.

There is a need to further explore the impact of Big Data technologies on the patient data models of hospital systems. Additionally, it was initially set out to test security and privacy of the interactive and functional BDA platform. However, due to the limitations of MapReduce, it was determined that its Java code would remain as is and it was determined not to add encrypted patient identifiers for personal health number, medical record number, and date of birth. Tang et al. [61] have implemented advanced indexed data of extralarge data sets with good performance after major adjustments to MapReduce programming. Further investigations need to not only test the use of MapReduce to encrypt the data, but also test querying the data afterwards on HBase.

## Figures and Tables

**Box 1:**
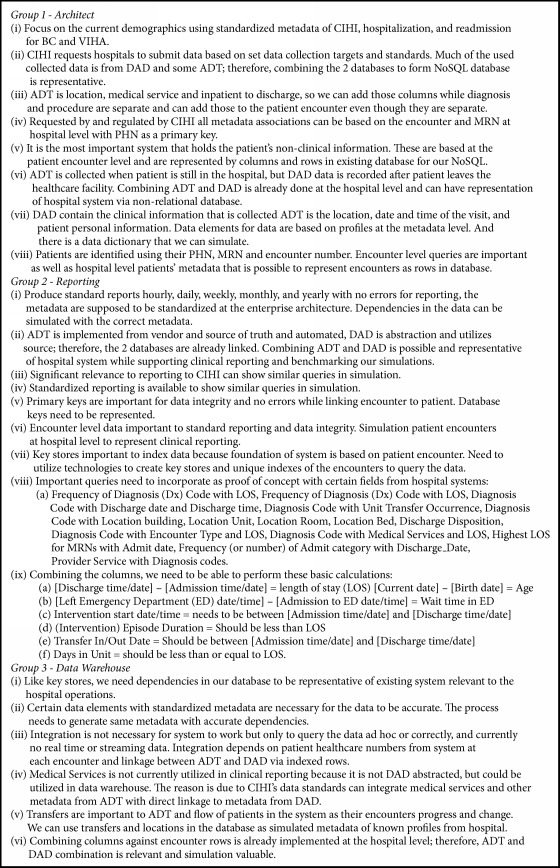
Information from interviewed groups involved in clinical reporting at Vancouver Island Health Authority (VIHA).

**Box 2:**
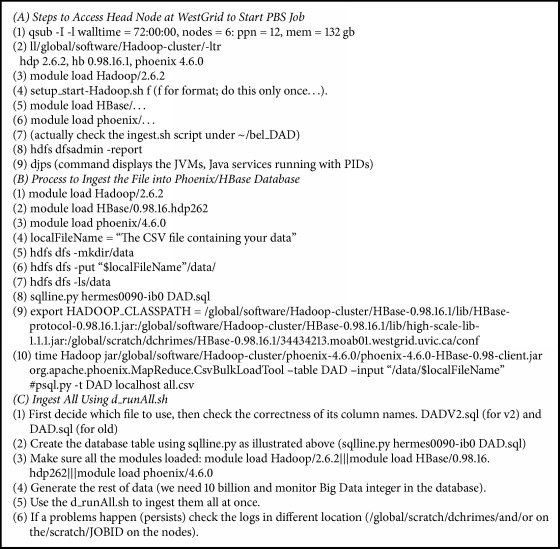
Configuration and command scripts run across BDA platform.

**Table 1:** Big Data applications related to clinical services [11–13, 18].

Clinical services	Healthcare Applications
R&D	(i) Targeted R&D pipeline in drugs and devices, clinical trial design, and patient recruitment to better match treatments to individual patients, thus reducing trial and failures and speeding new treatments to market, follow on indications, and discover adverse effects before products reach the market

Public health	(i) Targeted vaccines, e.g., choosing the annual influenza strains (ii) Identify needs, provide services, and predict patients at risk to prevent crises, especially for the benefit of populations

Evidence-based medicine	(i) Combine and analyze a variety of structured and unstructured data-EMRs, financial and operational data, clinical data, and genomic data to match treatments with outcomes, predict patients at risk for disease or readmission, and provide more efficient care

Genomic analytics	(i) Make genomic analysis a part of the regular medical care decision process and the growing patient medical record

Device/remote monitors	(i) Capture and analyze in real-time large volumes of fast-moving data from in-hospital and in-home devices, for safety monitoring and adverse prediction

Patient profile analytics	(i) Identify individuals who would benefit from proactive care or lifestyle changes, for example, those patients at risk of developing a specific disease (e.g., diabetes) who would benefit from preventive care

**Table 2:** Big Data technologies using Hadoop with possible applications in healthcare [5, 7–9, 11–13, 29, 37–42].

Technologies	Clinical utilization
Hadoop Distributed File System (HDFS)	It has clinical use because of its high capacity, fault tolerant, and inexpensive storage of very large datasets clinical.

MapReduce	The programming paradigm has been used for processing clinical Big Data.

Hadoop	Infrastructure adapted for clinical data processing.

Spark	Processing/storage of clinical data indirectly.

Cassandra	Key-value store for clinical data indirectly.

HBase	NoSQL database with random access was used for clinical data.

Apache Solr	Document warehouse indirectly for clinical data.

Lucene and Blur	Document warehouse not yet in healthcare, but upcoming for free text query on Hadoop platform, can be used for clinical data.

MongoDB	JSON document-oriented database has been used for clinical data.

Hive	Data interaction not yet configured for clinical data, but SQL layer to cross platform being possible.

Spark SQL	SQL access to Hadoop data not yet configured for clinical data.

JSON	Data description and transfer has been used for clinical data.

ZooKeeper	Coordination of data flow has been used for clinical data.

YARN	Resource allocator of data flow has been used for clinical data.

Oozie	A workflow scheduler to manage complex multipart Hadoop jobs not currently used for clinical data.

Pig	High-level data flow language for processing batches of data, but not used for clinical data.

Storm	Streaming ingestions were used for clinical data.

**Table 3:** Use cases and patient encounter scenarios related to metadata of patient visits and its database placement related to query output.

Case	Clinical Database
Uncontrolled type 2 diabetes & complex comorbidities	(i) DAD with diagnosis codes, HBase for IDs

TB of the lung & uncontrolled DM 2	(i) DAD and ADT columns with HBase for patient IDs

A on C renal failure, fracture, heart failure to CCU, and stable DM 2	(i) DAD and ADT columns with HBase for patient IDs

Multilocation cancer patient on Palliative	(i) DAD and ADT columns with HBase integrating data together

1 cardiac with complications	(i) DAD and ADT columns with HBase integrating data together

1 ER to surgical, fracture, readmitted category for 7 days and some complication after	(i) DAD and ADT columns with HBase integrating data together

1 simple day-surg. with complication, admitted to inpatient (allergy to medication)	(i) DAD and ADT columns with HBase for patient IDs

1 cardiac with complications and death	(i) DAD and ADT columns with HBase integrating data together

1 normal birth with postpartum hemorrhage complication	(i) DAD and ADT columns with HBase integrating data together

1 HIV/AIDS patient treated for an infection	(i) DAD and ADT columns with HBase for patient IDs

Strep A infection	(i) DAD and ADT columns with HBase integrating data together

Cold but negative Strep A. Child	(i) DAD and ADT columns with HBase integrating data together

Adult patient with Strep A. positive	(i) DAD and ADT columns with HBase for patient IDs

Severe pharyngitis	(i) DAD and ADT columns with HBase integrating data together

Child, moderate pharyngitis, throat culture negative, physical exam	(i) DAD and ADT columns with HBase for patient IDs

Adult, history of heart disease, positive culture for Strep A.	(i) DAD and ADT columns with HBase integrating data together

Adult, physical exam, moderate pharyngitis, positive for strep A. culture and positive second time, readmitted	(i) DAD and ADT columns with HBase for patient IDs

**Table 4:** Operational experiences, persistent issues, and overall limitations of tested Big Data technologies and components that impacted Big Data Analytics (BDA) platform.

Technology component	Clinical impact to platform
Hadoop Distributed Filing System (HDFS)	(i) Did not reconfigure more than 6 nodes because it is very difficult to maintain clinical data (ii) Had to add additional 2–4 TB for clinical data (iii) The clinical data needed large local disks

MapReduce	(i) Totally failed ingestion (ii) Clinical index files must be removed from node (iii) Extremely slow performance when working with clinical data (iv) Clinical data need more advanced algorithms

HBase	(i) *RegionServers* needed to form the clinical database (ii) Ongoing monitoring and log checking (iii) Run compaction (iv) Ran only 50 million rows of clinical data

ZooKeeper & YARN	(i) Extremely slow performance when ZooKeeper services are not running properly for both, but additional configuration minimized this limitation with few issues for YARN

Phoenix	(i) To maintain a database schema with current names in a file on the nodes, such that if the files ingested do not match, it will show error, and to verify ingested data exists within the metadata of schema while running queries (ii) This occurred zero times while ingesting files but many times at first when running queries

Spark	(i) Slow performance

Zeppelin	(i) 30-minute delay before running queries which takes the same amount of time as with Jupyter (ii) No fix to this issue

Jupyter	(i) Once the Java is established, it has high usability and excellent performance

Drill	(i) It is extremely fast but has poor usability (ii) Some integration to other interface engines
